# Supplementary material for: Photocatalytic and Cathode Active Abilities of Ni-Substituted α-FeOOH Nanoparticles
Source: Int J Mol Sci. 2023 Sep 19;24(18):14300. doi: 10.3390/ijms241814300 (PMC10532508; doi:10.3390/ijms241814300)
Supplement: Supplementary file 1 [file ijms-24-14300-s001.zip › ijms-2587771-supplementary.pdf]

Photocatalytic and cathode active abilities of Ni-substituted α-FeOOH nanoparticles

A. Ibrahim[1], M. Shiraishi[1], Z. Homonnay[2], E. Kuzmann[2], S. Krehula[3], M. Marciuš[3], A. Bafti[3], L. Pavić[3], S. Kubuki[1,*]

[1]Department of Chemistry, Graduate School of Science, Tokyo Metropolitan University, Tokyo, Japan

[2]Institute of Chemistry, Eötvos Loránd University, Budapest, Hungary

[3]Division of Materials Chemistry, Ruđer Bošković Institute, Zagreb, Croatia

*Corresponding author: kubuki@tmu.ac.jp

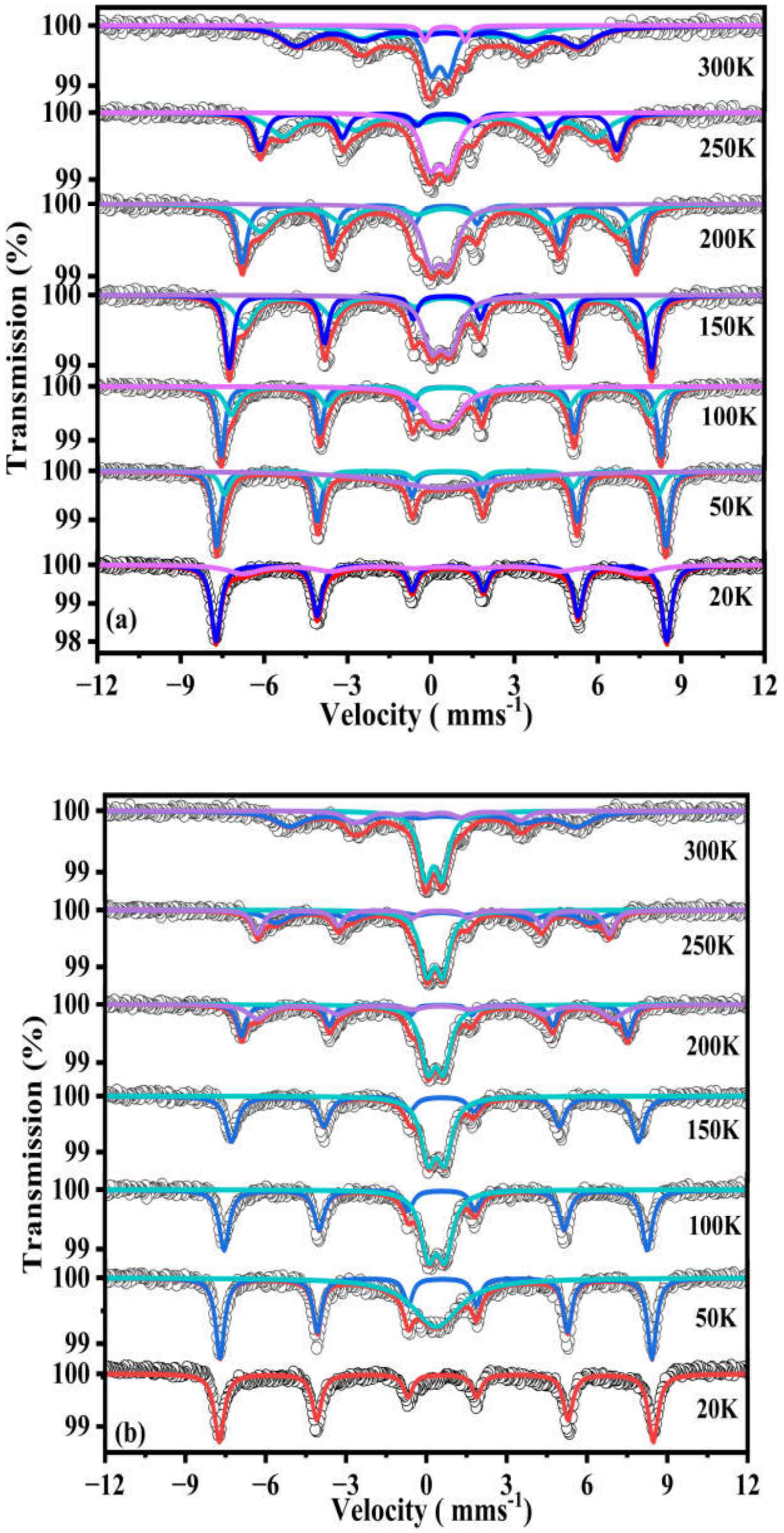

Figure S1. [57]Fe- Mössbauer spectra measured at low temperatures from 20K to 300K (a) Ni10 and (b) Ni20.

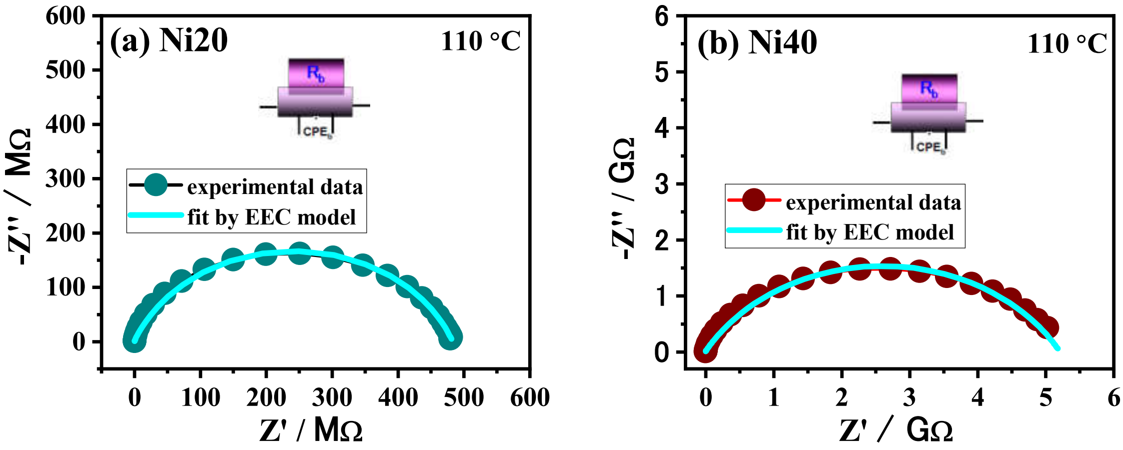

Figure S2. Complex impedance spectra @110 °C for sample (a) Ni20, and (b) Ni40. The symbols (colored circle) denote experimental values, whereas the solid cyan line corresponds to the best fit. The corresponding equivalent circuit model, comprised of parallel combination of the resistor (R) and the constant-phase element (CPE), used for fitting the data of an individual spectrum, and its interpretation is shown in each figure (defined as follows: b-bulk phase).

Table S1. Mössbauer parameters of α-$Ni_xFe_{1-x}OOH$ nanoparticles obtained from the ^{57}Fe-Mössbauer spectra measured in the range of velocity from 10 mm s^{-1} to -10 mm s^{-1} at 86K.

Sample	Component	A (%)	δ (mms^{-1})	Δ (mms^{-1})	Γ (mms^{-1})	H_{int} (T)	Phase
Ni0	sextet	80.50	$0.47_{\pm0.01}$	$-0.25_{\pm0.01}$	$(1\|6)0.43_{\pm0.01}$	32.9	Goethite
					$(2\|5)0.39_{\pm0.01}$		
					$(3\|4)0.34_{\pm0.04}$		
	sextet	19.50	$0.46_{\pm0.01}$	$-0.22_{\pm0.01}$	$(1\|6)0.28_{\pm0.04}$	50.1	Goethite
					$(2\|5)0.19_{\pm0.04}$		
					$(3\|4)0.35_{\pm0.16}$		
Ni5	sextet	71.39	$0.46_{\pm0.01}$	$-0.22_{\pm0.01}$	$(1\|6)0.39_{\pm0.01}$	49.6	Goethite
					$(2\|5)0.33_{\pm0.01}$		
					$(3\|4)0.35_{\pm0.02}$		
	sextet	20.42	$0.49_{\pm0.01}$	$-0.27_{\pm0.02}$	$(1\|6)0.50_{\pm0.06}$	47.8	Goethite
					$(2\|5)0.47_{\pm0.05}$		
					$(3\|4)0.38_{\pm0.08}$		
	doublet	7.65	$0.27_{\pm0.08}$	$0.00_{\pm10.11}$	$1.94_{\pm0.64}$	-	Unknown
Ni10	sextet	74.91	$0.47_{\pm0.01}$	$-0.24_{\pm0.00}$	$(1\|6)0.43_{\pm0.01}$	49.3	Goethite
					$(2\|5)0.37_{\pm0.01}$		

					**(3|4)0.33$_{\pm 0.01}$**		
	**doublet**	**12.63**	**0.46$_{\pm 0.01}$**	**0.66$_{\pm 0.03}$**	**0.53$_{\pm 0.09}$**	**-**	**Amorphous**
	**doublet**	**12.46**	**0.35$_{\pm 0.09}$**	**0.00$_{\pm 56.12}$**	**1.81$_{\pm 0.64}$**	**-**	**Unknown**
**Ni20**	**sextet**	**15.04**	**0.43$_{\pm 0.01}$**	**-0.10$_{\pm 0.03}$**	**(1|6)1.00$_{\pm 0.06}$**	**49.4**	**Goethite**
					**(2|5)0.82$_{\pm 0.05}$**		
					**(3|4)0.61$_{\pm 0.06}$**		
	**doublet**	**84.96**	**0.44$_{\pm 0.01}$**	**0.71$_{\pm 0.01}$**	**0.74$_{\pm 0.01}$**	**-**	**Amorphous**

δ: isomer shift, Δ: quadrupole splitting, Γ: FWHM, H_{int}: Internal magnetic field.

Table S2. Mössbauer parameters of α-$Ni_xFe_{1-x}OOH$ nanoparticles obtained from the ^{57}Fe-Mössbauer spectra measured in the range of velocity from 3 mm s^{-1} to -3 mm s^{-1} at 86 K

**Sample**	**Component**	***A* (%)**	**δ (mms^{-1})**	**Δ (mms^{-1})**	**Γ (mms^{-1})**	**H_{int} (T)**	**Phase**
**Ni20**	**sextet**	**12.37**	**0.43$_{\pm 0.01}$**	**-0.10$_{\pm 0.01}$**	**0.58$_{\pm 0.12}$**	**49.4**	**Goethite**
	**doublet**	**87.63**	**0.44$_{\pm 0.01}$**	**0.70$_{\pm 0.01}$**	**0.75$_{\pm 0.01}$**	**-**	**Amorphous**
**Ni50**	**doublet**	**36.80**	**0.45$_{\pm 0.01}$**	**0.89$_{\pm 0.01}$**	**0.45$_{\pm 0.01}$**	**-**	**Ferrihydrite**
	**doublet**	**63.20**	**0.44$_{\pm 0.01}$**	**0.47$_{\pm 0.01}$**	**0.39$_{\pm 0.01}$**	**-**	**Unknown**

δ: isomer shift, Δ: quadrupole splitting, Γ: FWHM, H_{int}: Internal magnetic field

Table S3: ^{57}Fe- Mössbauer spectra measured at low temperature from 20K to 300K of Ni10

T/K	Component	A (%)	δ (mms^{-1})	Δ (mms^{-1})	Γ (mms^{-1})	H_{int} (T)	Phase
**20**	**sextet**	**71.7**	**$0.48_{\pm 0.01}$**	**$-0.22_{\pm 0.01}$**	**$0.40_{\pm 0.01}$**	**$50.38_{\pm 0.02}$**	**Goethite**
	**sextet**	**28.3**	**$0.42_{\pm 0.02}$**	**$-0.13_{\pm 0.01}$**	**$1.38_{\pm 0.09}$**	**$44.25_{\pm 0.26}$**	**Goethite**
**50**	**sextet**	**45.9**	**$0.48_{\pm 0.01}$**	**$-0.22_{\pm 0.01}$**	**$0.33_{\pm 0.01}$**	**$50.27_{\pm 0.03}$**	**Goethite**
	**sextet**	**18.7**	**$0.50_{\pm 0.01}$**	**$-0.23_{\pm 0.02}$**	**$0.44_{\pm 0.03}$**	**$48.53_{\pm 0.11}$**	**Goethite**
	**doublet**	**35.5**	**$0.41_{\pm 0.04}$**	**$0.00_{\pm 0.02}$**	**$4.66_{\pm 0.58}$**	-	**Unknown**
**100**	**sextet**	**42.4**	**$0.46_{\pm 0.01}$**	**$-0.21_{\pm 0.01}$**	**$0.32_{\pm 0.01}$**	**$49.21_{\pm 0.02}$**	**Goethite**
	**sextet**	**30.2**	**$0.47_{\pm 0.01}$**	**$-0.26_{\pm 0.02}$**	**$0.58_{\pm 0.03}$**	**$46.78_{\pm 0.10}$**	**Goethite**
	**doublet**	**27.3**	**$0.37_{\pm 0.01}$**	**$0.63_{\pm 0.03}$**	**$1.11_{\pm 0.04}$**	-	**Unknown**
**150**	**sextet**	**37.1**	**$0.45_{\pm 0.01}$**	**$-0.22_{\pm 0.01}$**	**$0.36_{\pm 0.02}$**	**$47.22_{\pm 0.03}$**	**Goethite**
	**sextet**	**36.8**	**$0.46_{\pm 0.01}$**	**$-0.18_{\pm 0.02}$**	**$0.83_{\pm 0.03}$**	**$43.98_{\pm 0.12}$**	**Goethite**
	**doublet**	**26.1**	**$0.36_{\pm 0.01}$**	**$0.67_{\pm 0.02}$**	**$0.80_{\pm 0.03}$**	-	**Amorphous**
**200**	**sextet**	**38.1**	**$0.42_{\pm 0.02}$**	**$-0.24_{\pm 0.03}$**	**$1.07_{\pm 0.05}$**	**$40.01_{\pm 0.17}$**	**Goethite**
	**sextet**	**35.1**	**$0.42_{\pm 0.01}$**	**$-0.23_{\pm 0.01}$**	**$0.45_{\pm 0.02}$**	**$44.11_{\pm 0.04}$**	**Goethite**
	**doublet**	**26.8**	**$0.32_{\pm 0.01}$**	**$0.65_{\pm 0.02}$**	**$0.81_{\pm 0.03}$**	-	**Amorphous**

**250**	**sextet**	**42.7**	**$0.41_{\pm 0.01}$**	**$-0.24_{\pm 0.02}$**	**$1.19_{\pm 0.05}$**	**$34.95_{\pm 0.18}$**	**Goethite**
	**sextet**	**28.6**	**$0.40_{\pm 0.01}$**	**$-0.26_{\pm 0.01}$**	**$0.49_{\pm 0.02}$**	**$39.94_{\pm 0.05}$**	**Goethite**
	**doublet**	**28.7**	**$0.30_{\pm 0.01}$**	**$0.70_{\pm 0.02}$**	**$0.781_{\pm 0.03}$**	-	**Amorphous**
**300**	**sextet**	**47.8**	**$0.37_{\pm 0.06}$**	**$-0.26_{\pm 0.11}$**	**$1.37_{\pm 0.10}$**	**$31.55_{\pm 0.192}$**	**Goethite**
	**sextet**	**24.3**	**$0.47_{\pm 0.06}$**	**$0.00_{\pm 0.02}$**	**$1.15_{\pm 0.17}$**	**$18.85_{\pm 0.32}$**	**Unknown**
	**doublet**	**23.6**	**$0.31_{\pm 0.01}$**	**$0.63_{\pm 0.03}$**	**$0.61_{\pm 0.05}$**	-	**Amorphous**
	**doublet**	**4.4**	**$0.50_{\pm 0.02}$**	**$1.44_{\pm 0.04}$**	**$0.31_{\pm 0.07}$**	-	**Superparamagnetic**

δ: isomer shift, Δ: quadrupole splitting, Γ: FWHM, H_{int}: Internal magnetic field

Table S4. ^{57}Fe- Mössbauer spectra measured at low temperature from 20K to 300K of Ni20

**T/K**	**Component**	***A* (%)**	**δ (mms^{-1})**	**Δ (mms^{-1})**	**Γ (mms^{-1})**	**H_{int} (T)**	**Phase**
**20**	**sextet**	**100**	**$0.48_{\pm 0.01}$**	**$-0.22_{\pm 0.01}$**	**$0.43_{\pm 0.01}$**	**$50.37_{\pm 0.02}$**	**Goethite**
**50**	**sextet**	**54.9**	**$0.48_{\pm 0.01}$**	**$-0.24_{\pm 0.01}$**	**$0.39_{\pm 0.01}$**	**$50.14_{\pm 0.02}$**	**Goethite**
	**doublet**	**45.1**	**$0.36_{\pm 0.01}$**	**$-0.00_{\pm 0.01}$**	**$2.11_{\pm 0.12}$**	-	**Unknown**
**100**	**sextet**	**57.4**	**$0.47_{\pm 0.01}$**	**$-0.22_{\pm 0.01}$**	**$0.45_{\pm 0.01}$**	**$50.14_{\pm 0.02}$**	**Goethite**
	**doublet**	**42.6**	**$0.39_{\pm 0.01}$**	**$0.62_{\pm 0.01}$**	**$0.67_{\pm 0.02}$**	-	**Amorphous**
**150**	**sextet**	**55.9**	**$0.44_{\pm 0.01}$**	**$-0.25_{\pm 0.01}$**	**$0.53_{\pm 0.01}$**	**$47.20_{\pm 0.03}$**	**Goethite**
	**doublet**	**44.1**	**$0.37_{\pm 0.01}$**	**$0.61_{\pm 0.01}$**	**$0.66_{\pm 0.02}$**	-	**Amorphous**
**200**	**sextet**	**29.2**	**$0.44_{\pm 0.01}$**	**$-0.20_{\pm 0.03}$**	**$0.91_{\pm 0.06}$**	**$41.26_{\pm 0.23}$**	**Goethite**
	**sextet**	**28.9**	**$0.43_{\pm 0.01}$**	**$-0.23_{\pm 0.01}$**	**$0.40_{\pm 0.02}$**	**$44.83_{\pm 0.05}$**	**Goethite**
	**doublet**	**41.8**	**$0.35_{\pm 0.01}$**	**$0.59_{\pm 0.01}$**	**$0.64_{\pm 0.02}$**	-	**Amorphous**
**250**	**sextet**	**31.1**	**$0.41_{\pm 0.02}$**	**$-0.28_{\pm 0.04}$**	**$1.03_{\pm 0.07}$**	**$36.47_{\pm 0.26}$**	**Goethite**
	**sextet**	**27.7**	**$0.39_{\pm 0.01}$**	**$-0.24_{\pm 0.01}$**	**$0.48_{\pm 0.03}$**	**$40.82_{\pm 0.07}$**	**Goethite**
	**doublet**	**41.2**	**$0.31_{\pm 0.01}$**	**$0.60_{\pm 0.01}$**	**$0.60_{\pm 0.02}$**	-	**Amorphous**

**300**	**sextet**	**32.6**	**$0.35_{\pm 0.01}$**	**$-0.31_{\pm 0.03}$**	**$0.85_{\pm 0.05}$**	**$34.09_{\pm 0.12}$**	**Goethite**
	**sextet**	**27.0**	**$0.03_{0.05}$**	**$-0.49_{\pm 0.09}$**	**$1.43_{\pm 0.16}$**	**$24.10_{\pm 0.41}$**	**Goethite**
	**doublet**	**38.0**	**$0.29_{\pm 0.01}$**	**$0.60_{\pm 0.01}$**	**$0.56_{\pm 0.02}$**	-	**Amorphous**
	**doublet**	**2.5**	**$0.55_{\pm 0.02}$**	**$1.53_{\pm 0.02}$**	**$0.19_{\pm 0.05}$**	-	**Unknown**

δ: isomer shift, Δ: quadrupole splitting, Γ: FWHM, H_{int}: H_{int}: Internal magnetic field
